# Effect of Triticale Grain in Diets on Performance, Development of Gastrointestinal Tract and Microflora in Crop and Ileum of Broiler Chickens

**DOI:** 10.3390/microorganisms12061239

**Published:** 2024-06-20

**Authors:** Patrycja Wróblewska, Tomasz Hikawczuk, Anna Szuba-Trznadel, Andrzej Wiliczkiewicz, Andrii Zinchuk, Agnieszka Rusiecka, Krystyna Laszki-Szcząchor

**Affiliations:** 1 Department of Animal Nutrition and Feed Science, Wroclaw University of Environmental and Life Sciences, Chełmońskiego 38c, 61-630 Wroclaw, Poland; patrycja.wroblewska@upwr.edu.pl (P.W.); andrzej.wiliczkiewicz@upwr.edu.pl (A.W.); 2 Statistical Analysis Center, Wroclaw Medical University, Karola Marcinkowskiego 2-6, 50-368 Wroclaw, Poland; tomasz.hikawczuk@umw.edu.pl (T.H.); andrii.zinchuk@umw.edu.pl (A.Z.); agnieszka.rusiecka@umw.edu.pl (A.R.); krystyna.laszki-szczachor@umw.edu.pl (K.L.-S.)

**Keywords:** triticale, microorganisms, crop, broiler chickens, performance

## Abstract

The purpose of the research was to determine the effect of the use of a diet containing 30% triticale grain. In an experiment lasting 28 days, 180 one-day Ross-308 chickens (sex ratio 1:1) with an average initial body weight in treatment of 44.6 g were randomly assigned to 30 metabolic cages/replications, 6 birds in each. To compare the results between treatments, a one-way ANOVA was used with uneven replication numbers. The control group (I) received a standard diet containing maize and soybean meal. In the other treatments, 30% of different cereals were used: II—wheat, III—barley, and IV—triticale. Significant differences in body weight (BW) and feed conversion ratio (FCR) were observed on the 4th day of the life of broiler chickens (*p* < 0.05). Differences were determined between the control group (90.7 g BW and 1.32 kg of feed/kg BWG in the case of FCR) and birds receiving barley (93.0 g BW and 1.29 kg of feed/kg BWG in the case of FCR), compared to chickens fed diets with a 30% share of wheat grain (86.2 g BW and 1.53 kg feed/kg BWG in the case of FCR) and triticale (86.6 g BW and 1.53 kg feed/kg BWG in the case of FCR). Later, the differences in performance of birds between treatments did not occur (*p* > 0.05). In the nutrition of broiler chickens, control or 30% of the triticale diet caused a significant reduction (*p* < 0.01) of the number of *Escherichia coli* (*E. coli*) in the crop of broiler chickens (0 log cfu/g), compared to birds obtaining feed with 30% of wheat (1.78 log cfu/g). The diet containing triticale also reduced the number of *E. coli* (*p* < 0.05) within the ileum (0.78 log cfu/g) compared to chickens obtaining barley grain in the diet (2.12 log cfu/g). As a result of the use of triticale grain (*p* < 0.05), the total length of the bird intestines (199.64 cm) was compared to 30% of barley grain (209.76 cm). The increase in the length of the large intestine of broiler chickens in treatments was positively correlated (r = 0.613, *p* < 0.05) with the number of *Lactobacillus* sp. in the ileum. Triticale increased the pH in the crop of broilers chickens. The research results indicate that triticale, after longer storage, can be used in amounts of 30% of the diet without significant effect on the performance of broiler chickens, with a reduction in *E. coli* in crop in comparison with wheat and in ileum with barley.

## 1. Introduction

Triticale is a species of cereal bred in Scotland and Germany as a result of crossing wheat and rye varieties [1]. Therefore, it combines the features of these two cereal grains: High content of crude protein and digestible energy in wheat and high yield and biological value of rye [2]. It is characterized by a similar level of metabolic energy to wheat and corn [3]. Triticale contains more total protein than corn or rye. Additionally, better quality protein in terms of the content and proportion of exogenous amino acids for monogastric animals [4,5].

Its use on a global scale is small, but due to better yields, it is possible to use it in the nutrition of monogastric animals as feed produced on farms, especially on acidic soils and with lower quality classes [2]. Triticale can be a valuable feed for local farms [6]. Due to the need to use a small amount of fertilizers and pesticides and to limit soil erosion, it is a valuable raw material in organic and sustainable production [4,7]. Of course, the selection of the variety is of great importance in this case (the share of rye and wheat genes), which will consequently translate into nutritional value and yield in specific conditions [8].

Triticale does not show such a beneficial increase in the body weight of chickens compared to corn grain. However, due to its high energy content, it is possible to partially replace corn with triticale in the chicken diet without significant deterioration of production parameters as well as the quality of poultry meat [9,10].

A characteristic feature of triticale grain (as well as the two species on which it is based) is the content of non-starch polysaccharides and their action in the digestive tract. These compounds increase the viscosity of food content, which consequently limits the digestion and absorption of nutrients [11,12]. Young animals that do not yet have a fully mature digestive tract are most exposed to their adverse effects [13]. Triticale grain is characterized by a high content of arabinoxylans, anthocyanins, and phenolic acid, which in small amounts have probiotic and antioxidant properties, increasing the number of microflora in the cecum [14,15,16]. In larger amounts, pentosans and beta-glucans negatively affect the digestive processes and absorption of nutrients [17]. Polish nutritional recomendations provide for the use of triticale at the level of 10% of the diet of broiler chickens normally and 30% after supplementing with an enzyme preparation [18]. Higher amounts, from 20–40%, due to anti-nutritional substances have a negative effect on the deterioration of bird performance [19]. The process of storing grains and longer storage of more than three months has a positive effect on reducing their content and thus improving the performance of chickens, decreasing the viscosity of digesta, and improving nutrient digestibility [20]. On the other hand, the quality of grain also decreases with storage time [21].

Depending on the composition of the diet of broiler chickens, the weight of organs and the length of individual intestinal sections may change [22]. Additionally, like corn, wheat, or barley, triticale also modifies the composition and development of the gastrointestinal microbiome [23,24]. Scientific journal databases contain little information on the effect of triticale on the microflora of the gastrointestinal tract. Santos et al. [10] observed a lack of colonization of the ileum by *Salmonella* sp. bacteria after previous inoculation of chickens with *Salmonella enterica* bacteria. The high proportion of triticale grain (60%) also increases the number of *Coliforms* and reduces *Lactobacillus* sp., while the addition of feed enzymes causes the opposite effect [25]. Widodo et al. [26] also draw attention to the fact that the use of different varieties in Australian conditions has an impact on the differentiation of treatments with the addition of phytase and xylanase in the case of *Clostridium perfingens* and lactic acid bacteria (LAB) in the ileum and Enterobacteria in the ceca.

The aim of the study was to determine the impact of a diet containing 30% triticale grain stored for four months and to compare the results obtained with similar proportions of wheat and barley grain in the diet and a control mixture based on maize and soybean meal. The analyzed effect includes production parameters, weights and lengths of selected sections of the digestive tract, and the composition of the microflora of the crop and ileum, as well as determining the occurrence of a correlation and its intensity between the weights of organs and intestinal sections and the number of selected microorganisms in the crop and ileum.

## 2. Materials and Methods

### 2.1. Chickens, Diets, Nutrition and Conditions

The research was approved by the Local Ethical Committee for Animal Experiments in Wroclaw as part of its own research at the Wroclaw University of Environmental and Life Sciences (protocol no. 084/2010). The experiment was conducted on 180 chickens kept in 30 metabolic cages in a one-factorial design. The differentiating factor was the type of cereal grain in the diet (Table 1). Vaccination was conducted during the ovo phase against Gumboro disease and after the hatching of chickens against infectious bronchitis.

The control group consisted of maize and soybean meal (7 replications). In the second group, 30% wheat grain was used in the diet (7 replications). In the third and fourth, 30% of barley and triticale grains were used, respectively (8 replications each). Each replication contained 6 observations/animals, on the basis of which the mean value within it was determined. In the experiment, 180 Ross-308 chickens were randomly assigned to four different treatments. The body weight of chickens in treatments was equal and amounted to approximately 44.6 g. The birds were kept on a starter diet until the 28th day of life. Except for the control group, in the rest of the treatments, maize was replaced with 30% wheat, barley, or triticale.

The European Poultry Energy Tables [27] (determining the level of metabolic energy in feed components based on chemical composition) and the Polish Recommendation of Poultry Nutrition [18] were used to prepare individual diets with the required level of nutrients. The mixtures were composed in order to achieve a content of metabolic energy and total protein close to 12.5 MJ/kg and 220 g, respectively. Chickens received complete mixtures in loose form, *semi ad libitum*, in feeders. During the experiment, the number of uneaten meals was recorded. Water was administered using a nipple system.

The chickens were kept under standard conditions consistent with the breeding program for Ross-308. The temperature on the first day of rearing was 33 °C, it was then systematically lowered to 20 °C on the last day. The lighting program in the first 10 days included 20 h of light and 4 h of darkness in the hen house. Then, from days 11 to 20, 18 h of light and 6 h of darkness were included. After day 21, the light was on for 16 h and turned off for 8 h. The humidity in the room ranged from 60 to 72%.

### 2.2. Performance of Broiler Chickens

On the first day of rearing, the chickens were randomly assigned to 30 replications with a similar body weight of 46.6 g ± 0.3 g. Individual treatments showed homogeneity in terms of variance. Body weight (BW) was determined on 4, 18, and 28 days of life. Feed intake (FI) was also recorded on the same days. Feed conversion ratio (FCR) in a cumulative form from days 1–4, 1–18, and 1–28. During the experiment, the amount of feed uneaten by the chickens was determined and included in the FCR calculation. Mortality in the groups was low and did not exceed 3%.

### 2.3. Component and Chemical Analysis

AOAC (2005) methods [28] were used to determine the composition of feed components and diets. In the Weenden analysis, the content of dry matter (DM, AOAC; 934.01), total protein was determined using the Kjeldahl method (CP, AOAC; 984.13), crude ash (CA, AOAC; 942.05), and crude fat using the Soxhlet method (EE, AOAC; 920.39A), crude fiber using the Hennenberg and Stohman method (CF, AOAC; 978.10). Values for nutrients in components and diets were determined in the laboratory. The metabolic energy content of the diets was calculated based on equations from the European Energy Tables for Poultry [27].

### 2.4. Dissection of Gastrointestinal Tract of Birds

On the 28th day of the birds’ lives, when the state of the gastrointestinal microflora was stabilized, 90 broiler chickens were sacrificed by concussion following a truncheon strike and a quick sublingual incision for the bleeding of birds. From the foregut: crop, proventriculus, and gizzard were dissected and weighted after emptying from the digesta. In the case of the hindgut: measurements were conducted after emptying the digesta: duodenum, jejunum, ileum, ceca, large intestine, and sum of all sections as the total length of the intestines. Measurements were taken continuously during the dissection.

### 2.5. Determination pH Value in Crop and Ileum

In the digesta of crop and ileum, the pH value was determined. For this purpose, pooled samples were collected from three birds from each replicate. After collection, the research material within the replication was mixed together and analyzed using a CP-401 Elmetron pH meter (Elmetron Company, Zabrze, Silesian Voivodeship, Poland). Before starting the analysis, the pH meter was calibrated. Then, in each trial, the pH value was measured three times. After each measurement, the electrode was washed with distilled water. In the end, the measurement values were used to calculate the mean value from the replication, which was included in the calculation of the mean value from treatment.

### 2.6. Microbiological Analysis

During the dissection, the digestive tract of the chickens was measured, and the crop and a section of the ileum (10 cm from the part proximal to the Meckel’s diverticulum) were collected from the chickens for microbiological analysis on the 28th day of the birds’ lives. The collected samples were packed into separate sterile zip-lock bags and transported immediately from the poultry house to the laboratory for microbiological analysis at ambient temperature. The plate method was used for microbiological analysis, whereby individual groups of microorganisms were inoculated using selective culture-based plate techniques and incubated. In the laboratory, using this method to determine the total aerobic microbial count (TAMC), total yeast and mold count (TYMC), the number of *Escherichia coli* (*E. coli*), *Salmonella* sp., and *Lactobacillus* sp. After this process, the quantity of microorganisms was specified in cfu/g. These procedures were identical to those described by Wróblewska et al. [24].

### 2.7. Statistical Analysis

All obtained data were prepared in Excel in a form that can be used in statistical programs. Data from individual replications were taken into account in the form of a mean for replication, and then the mean value for the entire treatment was determined. Additionally, instead of standard deviations for each treatment, SEM was determined for 30 replications. All data were statistically verified using a one-way analysis of variance. However, data on the weights of selected organs and intestinal length were used using one-way analysis of covariance, where the covariate was the final body weight of chickens. The Tibco Statistica 13.3 software program (Tibco Software Inc., Palo Alto, CA, USA) was used for analysis [29]. The normality of the data distribution was verified using the Shapiro–Wilk test. Equality of variances were determined using Leven’s test. Differences between treatments were determined using the Tukey test (uneven number of replications in treatments). Statistical significance was determined at two levels: *p* < 0.05 and *p* < 0.01. Additionally, the correlation coefficient (r) was determined between the weight of organs, the length of intestinal sections, and the number of microorganisms in the crop and ileum. The statistical significance of the correlation was considered at *p* < 0.05.

## 3. Results

### 3.1. Performance of Broiler Chickens

In the case of BW and BWG, significant differences between groups were observed on the 4th day of rearing (*p* < 0.05, Table 2). Chickens fed a diet containing barley obtained higher (*p* < 0.01) BW and BWG (93.0 g and 48.3 g, respectively) in comparison with wheat (86.2 g and 41.3 g, respectively) and triticale (86.6 g and 41.9 g, respectively). The control diet also differs significantly from wheat and triticale, but at a lower significance level (*p* < 0.05).

On the remaining dates, the differences observed were not statistically significant (*p* > 0.05). In the case of feed intake, no significant differences (*p* > 0.05) were found between treatments. FCR differentiated in the first period of chick life, 1–4 days of age (*p* < 0.05). The lowest values were recorded in the control group and chickens receiving a diet containing barley grain (1.32 and 1.29 kg of feed/kg of BWG, respectively). The highest was in the case of the wheat and triticale groups (1.53 kg of feed/kg of BWG, respectively). Later, no significant differences were found (*p* > 0.05).

### 3.2. The Weight of Organs and the Length of the Intestines of the Digestive Tract Depending on the Type of Diet

The final body weight was significantly different between treatments (*p* > 0.05). Therefore, in the further analysis of covariance, the final weight was a covariate for comparing individual components of the digestive tract of chickens in a reference system. There was a significant effect (*p* < 0.05, Table 3) on crop weight. In other cases, no significant effect on the results of treatments was stated. The lowest empty crop weight was found in the treatment fed with wheat grain (5.46 g). This group, however, showed significant differences with the control and chickens fed the barley diet (6.53 and 6.52 g, respectively). A significantly higher glandular stomach weight (*p* < 0.01) was found in chickens fed a barley grain diet compared to the group of chickens fed a wheat diet (6.09 and 5.50 g, respectively). The observed relationships were similar to those in the case of the muscular stomach (*p* < 0.05, obtaining 24.72 and 22.17 g, respectively). There was no difference in the length of the duodenum and small intestine depending on diet (*p* > 0.05). In the case of the length of the cecum, a highly significant difference was found between chickens fed a diet containing barley grain compared to triticale (35.19 and 30.90 cm, respectively).

In the large intestine, significant differences were found at two levels of significance: Between chickens on barley and wheat diets (*p* < 0.01, 7.68 and 5.69 cm, respectively) and between groups receiving diets based on barley and corn and post-extraction soybean meal (control group) (*p* < 0.05, 7.68 and 6.28 cm, respectively). In the case of the total length of the intestines, the barley diet also significantly increases their length compared to the group of chickens receiving triticale grain in the diet (209.76 and 199.64 cm, respectively).

### 3.3. Count of Microorganisms and pH Value in Crop and Ileum

**Figure 1:**
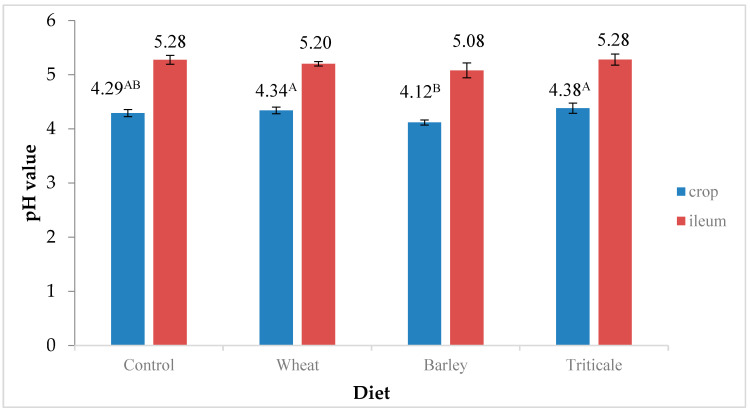
Effect of diet type on the pH of crop and ileum. Means in labels with different superscripts A, B significant at *p* < 0.01.

Figure 1 shows the pH values in the crop and ileum. A diet containing barley grain significantly reduces (*p* < 0.01) the pH of crop content (4.12) compared to wheat and triticale grains (4.34 and 4.38, respectively). In the ileum, no statistically significant differences were found in the pH of the content (*p* > 0.05).

Table 4 shows significant differences in terms of individual diets. The highest value was found for TAMC in the control group (6.34 log cfu/g), followed by triticale (5.26 log cfu/g), barley (4.63 log cfu/g), and wheat (4.11 log cfu/g). *E. coli* bacteria were not detected in the control group and those receiving triticale grain in the diet, which differed significantly (*p* < 0.01) from chickens receiving wheat grain in the diet (1.78 log cfu/g). The highest content of *Lactobacillus* sp. (*p* < 0.01) was found in the control group receiving corn grain in the diet (3.35 log cfu/g) compared to the mixture containing triticale (2.93 log cfu/g). The other two diets contain wheat and barley. They contained even less (*p* < 0.01) compared to the remaining groups of *Lactobacillus* sp. bacteria (2.51 and 2.22 log cfu/g, respectively).

In the case of molds and yeasts, a significant difference (*p* < 0.01) was found between the control group (2.39 log cfu/g) and chickens fed a diet containing barley grain (2.05 log cfu/g). There were no statistical differences (*p* > 0.05) between the remaining groups and those mentioned above. There were no statistical differences within the TAMC in the ileum (*p* > 0.05). A significant increase (*p* < 0.05) in the number of *E. coli* was found in the ileum of chickens fed a diet containing barley grain (2.12 log cfu/g). In turn, the participation of triticale in the diet significantly reduced (*p* < 0.05) the number of *E. coli* in this section of the digestive tract (0.78 log cfu/g). The use of barley grain in the diet of chickens increased (*p* < 0.01) the number of *Lactobacillus* sp. compared to other diets. Wheat grain in the diet reduced significantly (*p* < 0.05) the number of molds and yeasts (0.45 log cfu/g) compared to wheat grain (1.96 log cfu/g).

### 3.4. Correlation between the Weight of Organs and the Length of the Intestines and the Number of Microorganisms in the Crop and Ileum

Analyzing the correlations between the variables, weak relationships were found in most cases, with r values ranging from −0.490 to 0.613 (Table 5). In the case of bacteria found in crops, a significant, weak positive correlation was found between crop weight and TAMC (r = 0.467). However, a weak negative correlation occurred between the weight of the crop and proventriculus and the number of *E. coli* in the crop (r = −0.490 and r = −0.406, respectively). There was no significant relationship between the variables in the case of the size of the stomach and duodenum (*p* > 0.05). It was also found that the increase in the length of the jejunum had a positive effect on TAMC in the crop (r = 0.401). However, negative correlations in the number of *E. coli* and *Lactobacillus* sp. were determined in the case of growth of the large intestine (r = −0.425) and cecum (r = −0.407), respectively. Moreover, the increase in the length of the ileum, cecum, and large intestine is negatively correlated with TYMC in the range from r = −0.418 to r = −0.456.

Analyzing the count of microorganisms in the ileum, there were no significant relationships (*p* < 0.05) between the size or length of organs and changes in TAMC and *E. coli*. As the proventriculus and gizzard increased, so did the number of *Lactobacillus* sp. (r = 0.482 and r = 0.490, respectively). Moreover, the number of *Lactobacillus* sp. was also positively correlated with the increase in the length of the cecum (r = 0.449) and large intestine (r = 0.613). A negative correlation in the case of TYMC was found with increasing duodenal length (r = −0.391).

## 4. Discussion

### 4.1. The Influence of Triticale on Production Parameters

The mixtures used in the experiment were isoprotein and isoenergetic. The use of different cereal grains resulted in the diversification of diets in terms of crude fiber. Throughout the entire experimental period until the 28th day of life, the chickens received starter diets. The use of 30% of three different cereal grains in treatments, apart from the control group, did not significantly affect the production parameters at the final stage of the experiment. From the point of view of triticale, this type of phenomenon was certainly influenced by the longer grain storage time (4 months after harvesting). Typically, the use of more than 10% triticale requires the use of enzymes in the form of xylanases, which limit the anti-nutritional effect of arabinoxylans contained in grain [30].

The BW of one-day-old chicks was equal at the very beginning of the experiment. Variation in body weight was demonstrated on the 4th day of life. A slight increase in the crude fiber content by using 30% barley in the mixture significantly improved the digestibility of nutrients, similarly to the control mixture, which could be due to the high proportion of corn. In which the outer layer of the grain shows greater hardness than in the case of wheat or triticale [31]. After this period, the influence of non-starch polysaccharides, especially arabinoxylans, was no longer so significant. Additionally, the effect of compensation for the growth of chickens was noted in the remaining groups, so no significant differences were found on days 18 and 28 in BW (*p* < 0.05). In their meta-analysis, Ditengou et al. [19] indicate that triticale may have different effects on the production parameters of broiler chickens. On the one hand, the share of 20 to 40% of this grain has a similar effect as the control diet, which may be largely related to the storage time of such grain and a significant reduction in the share of non-starch polysaccharides (NSP). However, Osek et al. [32] determined in their study the negative impact of replacing wheat with triticale in the case of BW and FCR. Kliševicūtė et al. [17] also noticed in their experiment worse body weight results throughout the rearing period in relation to triticale when it was used in a proportion from 2 to 25%.

Moreover, the experiment conducted by Witzig et al. [33] indicates a higher digestibility of phosphorus contained in triticale grain compared to wheat grain, and increasing the level of triticale grain in the diet does not require the use of phytase. However, if the grain is used for a short period after harvest, it is worth limiting the effect of the soluble NSP contained in triticale grain to more than 20%.

In the experiment, feed intake did not show significant differences in the intervals from 1–4, 1–18, and 1–28 days of age. The difference between groups in feed efficiency was observed from 1–4 days (*p* < 0.05). Later, no significant differences in this production index were found (*p* > 0.05). In nutritional practice, if triticale constitutes more than 10% of the concentrate mixture, enzymes containing mainly xylanase are added. However, the problem with the high share of NSP in the diet and their anti-nutritional effect occurs mainly up to the 10th day of life [18]. In the study by Wiśniewska et al. [34], which reported the effect of using xylanase individually on the improvement of BWG with a lower FI and, consequently, FCR, compared to the control group in the first 11 days of the experiment, the application of an emulsifier into a diet of broiler chickens improved BWG. On the other side, Zarghi and Golian [9] in their research, replacing maize grain with triticale in the percentage from 0 to 100%, did not find significant differences in production parameters between the 4th and 10th days of rearing (BWG, FI, and FCR). They noticed a significant increase in FI and FCR in broiler chickens (*p* < 0.05) when maize was completely replaced with triticale compared to the control diet between days 11–28 of bird rearing. A lower percentage of triticale did not result in significant differences in results compared to the control.

In the conducted experiment, a positive effect in the first days on performance was observed for a diet containing 30% barley, which suggests that a small addition of insoluble fiber to the diet (oat hull) may also reduce the level of soluble NSP in the diet by dilution effect [35]. Additionally, Bornaei et al. [36] pointed out in their research that one of the ways to reduce the negative effects of non-starch polysaccharides contained in barley grain is electron beam irradiation. In these studies, a 50% share of barley grain in the diet subjected to this process resulted in higher BWG and FI in the first 21 days of rearing.

### 4.2. The Weight of Organs and the Length of Intestinal Sections Depending on the Type and Share of Cereals in the Diet

Among the individuals randomly selected for slaughter, significant differences in slaughter weight were found (*p* < 0.05); therefore, the statistical analysis was performed using one-way analysis of covariance, where the covariate for a given weight or length measurement was the final body weight. Wheat and triticale in the diet resulted in a lower empty crop weight compared to corn, which had a higher grain hardness (*p* < 0.05). However, a higher share of crude fiber in barley grain resulted in a significant difference in the empty weight of the crop compared to the group of chickens fed wheat (*p* < 0.05). In the case of the diet containing triticale or barley, there were no differences in the weight of empty crops (*p* > 0.05).

The presence of crude fiber in a diet containing only 30% barley resulted in a significant increase in the mass of the muscle gizzard (*p* < 0.01) in this group. Similar conclusions were obtained in the study of Viliene et al. [37], where an increasing share of barley (from 6 to 25% in kg of the mixture) in the diet of chickens from the 1st to the 5th week resulted in an increase in the mass of the muscle gizzard compared to the control group and a low share of barley in the diet. In the experiment, triticale grain stimulated an increase in the mass of the muscle gizzard at a similar level as in control group chickens. The share of rye genes resulted in a higher weight of this organ compared to the wheat grain. Similar relationships were noted in the case of the glandular stomach, where the differences were significant (*p* < 0.05).

There was no significant difference (*p* > 0.05) in the length of the duodenum and small intestine sections, which suggests that the share of NSP did not have a significant impact on the process of digestion and absorption of nutrients and the related increase in the length of the intestines in the case of 50% and higher shares. In the study of Viliene et al. [38], when using low and high levels of barley grain in the diet, the percentage of intestinal content was lower (*p* < 0.05) compared to the control group and medium level of barley in the diet, suggesting that the proportion of barley in the amount of 6 to 20% released more nutrients, which was accompanied by an increase in the percentage of intestines in live weight.

Triticale grain in the diet significantly shortened the length of the cecum compared to barley grain. In the case of the large intestine, the barley diet also increased the length of this section of the intestine. In terms of values, triticale increased in length compared to the control and wheat grain diets. Additionally, the total length of the intestines was significantly lower in the case of a diet based on triticale grain, compared to barley grain, which contains a higher proportion of crude fiber, mainly insoluble dietary fiber [38]. Furthermore, Kouzunis et al. [15] indicate an increase in the role of the muscular stomach and an increase in the digestibility of nutrients, which may also be caused by an increase in the length of the intestines and a reduction in the viscosity of the chyme.

Triticale influenced the highest pH values in the crop and small intestine. This may suggest the use of whole grain later in bird rearing or oat hulls to increase the secretion of hydrochloric acid by the glandular stomach [39,40]. Additionally, Singh et al. [41] indicate in their research that it is possible to effectively reduce the pH of the muscle stomach by using the pellet process of feed, but, as in the case of the experiment, no significant differences in pH were noted in the ileum; however, this may have been due to a higher inter-group variability, especially in groups using barley and triticale.

### 4.3. Number of Bacteria in the Crop and Small Intestine

The stability of the animal microbiome is one of the basic conditions in their rearing, affecting health and production parameters, and its composition is most influenced by the types of components in the diets used [42]. In the case of broiler chickens, microbiome differentiation occurs already during embryonic development and in the first two weeks after hatching [43,44]. Additionally, Dittoe et al. [45] draw attention to the fact that the composition of microflora depends on many factors, mainly: age, environment, and feed composition.

Karimi et al. [46] also point to the fact that the composition of *Lactobacillus* sp. in the microbiome is relatively stable, and only an increase in the share of pathogenic bacteria as a result of their presence in the feed or environment may cause microbiome disorders. In the conducted research, triticale increased the number of TAMCs compared to wheat and barley. Moreover, the number of TAMCs in the crop was positively correlated with its weight.

Among all groups, triticale was characterized by a reduction in the number of *E. coli* both in the crop and in the small intestine, which may also reduce the occurrence of colibacillosis in chickens. The reduction in *E. coli* was associated with an increase in the weight of the crop and glandular stomach. Bornaei et al. [36] reported a significant decrease in the number of *E. coli* bacteria in the ileum of broiler chickens after electron treatment beam irradiation for barley grain, which also suggests the possibility of using this treatment for wheat and triticale grain. When the xylanase enzyme was used in chickens receiving wheat grain in their diet, Wang et al. [47] found an increase in the number of bacteria of the genus *Lactobacillus* and *Bifidobacterium* and bacteria producing butyric acid in the fermentation process. The count of bacteria of the genus *Ruminococcae* and *Bacterioidetes* decreased with the amount of NSP. Barley grain up to 20% also significantly increased the level of butyric acid in the cecum [37].

Triticale grain also showed an increase in the number of *Lactobacillus* bacteria sp. The increase in the number of these bacteria in the small intestine was associated with an increase in the mass of the glandular and muscular stomach. This may, to some extent, interact with the initial preparation of food content for digestion in the further part of the digestive tract and be related to the easier degradation of arabinoxylans compared to wheat grain into XOS, showing a prebiotic effect [48]. Additionally, Bedford [49] described the positive impact of NSP-degrading enzymes on the microbiome of the ileum and caecum. Stanley and Bajagal [50] observed in the conducted experiment that the *in ovo* administration of the *Lactobacillus agilis* strain influenced the rapid establishment and stabilization of the chicken microbiome, which allows for rapid colonization of the gastrointestinal tract in the first week of life. However, the type of diet also plays an important role in stabilizing the microbiome and reducing the occurrence of inflammation. In the experiment, an increase in the length of the cecum decreased the number of these bacteria in the crop (*p* < 0.05, r = −0.407), while the increase in the length of the large intestine increased the number of *Lactobacillus* sp. in the ileum (r = 0.613). Moreover, research by Munyaka et al. [51] shows that when enzymes in the form of xylanase and β-glucanase are used, they do not influence the occurrence of changes at the level of individual phyla, while changes in the composition of microorganisms take place at lower units of taxa. The lack of changes may also be due to the stabilization of the microbiome on the 21st day of life; hence, the use of enzymes may not have had such a significant impact on the differentiation of the microbiome. ileum and caeca [52].

The use of triticale increased the number of TYMCs in the small intestine compared to other diets. The increase in duodenal length was negatively correlated with TYMC in the small intestine. Olson et al. [53] point out that an increase in the level of molds in the ileum may be associated with an increase in the concentration of mycotoxins; therefore, if triticale grain is used, the diet can be supplemented with sorbents of these substances. In the tested experiment, no *Salmonella* bacteria were found in the samples tested either in the crop or in the initial section of the ileum, constituting a threat to the processing chain.

## 5. Conclusions

The use of triticale grain in 30% of the diet did not affect significant differences in the performance of broiler chickens on the 28th day of life. Differences in body weight and FCR were found on the 4th day of life. Later, due to the mechanisms of compensation and microbiome stabilization, differences were not significant. The use of triticale in the diet reduced the count of *E. coli* in the crop and ileum. Moreover, as a result of its use, the total length of the intestines in broiler chickens was shortened. Triticale influenced the increase in pH, which suggests that the addition of sources of insoluble dietary fiber stimulated the development of gizzards and an increase in the production of hydrochloric acid. That possible change in the number of TYMC within the ileum and a decrease in the effect of NSP in the diet in the first week of broiler chicken rearing. In the conducted experiment, the increase in the length of the large intestine was accompanied by an increase in the number of *Lactobacillus* sp. in the ileum, which, from a physiological point of view, was caused mainly by the presence of barley in the diet. The experiment results indicate that triticale, after longer storage, can be used in amounts of 30% of the diet without significant effect on the growth performance of broiler chickens, with a reduction in *E. coli* in crop in comparison with wheat and in ileum with barley.

## Figures and Tables

**Table 1:** Ingredient and chemical composition of diets.

Item	Control	Wheat	Barley	Triticale
*Ingredient composition*				
Corn	56.17	26.33	23.03	27.56
Wheat		30.0		
Barley			30.0	
Triticale				30.0
Rape oil	3.0	4.1	5.8	4.2
Soybean meal (46% CP)	36.9	35.7	37.4	34.3
Dicalcium phosphate	2.15	1.99	1.91	2.03
Limestone	0.14	0.22	0.27	0.23
NaCl	0.34	0.35	0.35	0.35
L-Lysine HCl	0.084	0.095	0.025	0.109
DL-Methionine	0.216	0.215	0.215	0.221
Premix *	1.0	1.0	1.0	1.0
*Calculated analysis*				
Metabolizable energy, MJ/kg	12.54	12.55	12.51	12.56
*Analyzed nutrients*				
Crude protein, g	225.5	227.1	226.8	224.9
Crude fibre, g	26.8	28.3	34.2	26.7
Ca, g	6.49	6.50	6.50	6.49
P_available_, g	4.30	4.29	4.30	4.30
Na, g	1.58	1.59	1.59	1.59
Lysine, g	12.03	11.99	12.04	12.02
Methionine, g	5.50	5.50	5.49	5.51

* Premix provided per kilogram of the diet following amounts of vitamins and minerals: vitamin A 10,000 IU, vitamin D_3_ 2000 IU, vitamin E 20 mg, vitamin K 3 mg, vitamin B_1_ 2.5 mg, vitamin B_6_ 0.4 mg, vitamin B_12_ 0.015 mg, nicotonic acid 60 mg, pantothenic acid 8 mg, folic acid 1.2 mg, choline chloride 450 mg, DL-Methionine 1.0 mg, Mn 74 mg, Fe 30 mg, Zn 45 mg, Cu 4 mg, Co 0.4 mg, I 0.3 mg.

**Table 2:** Performance of broiler chickens.

Item	Treatment				SEM	*p*-Value
	Control	Wheat	Barley	Triticale		
*BW, day*						
4	90.7 ^a^	86.2 ^Bb^	93.0 ^Aa^	86.6 ^Bb^	0.747	0.000
18	581.2	576.5	574.3	562.3	3.121	0.138
28	1231.3	1235.7	1265.3	1225.9	6.788	0.120
*BWG, days*						
1–4	45.9 ^a^	41.3 ^Bb^	48.3 ^Aa^	41.9 ^Bb^	0.721	0.000
1–18	536.4	531.7	529.6	517.6	3.012	0.141
1–28	1186.5	1190.9	1220.7	1180.9	6.561	0.119
*FI, days*						
1–4	60.5	63.1	62.1	64.0	0.605	0.184
1–18	882.6	891.0	876.9	891.4	8.962	0.926
1–28	1772.6	1757.3	1759.8	1777.4	11.60	0.912
*FCR, days*						
1–4	1.32 ^B^	1.53 ^A^	1.29 ^B^	1.53 ^A^	0.030	0.000
1–18	1.65	1.68	1.66	1.72	0.021	0.559
1–28	1.49	1.48	1.44	1.51	0.012	0.242

Means in the same row with different superscripts A, B significant at *p* < 0.01; a, b at *p* < 0.05.

**Table 3:** Organ weight and intestinal length depending on the type of diet.

Item	Treatment				SEM	*p*-Value
	Control	Wheat	Barley	Triticale		
Live weight *	1.30 ^a^	1.27 ^ab^	1.27 ^ab^	1.23 ^b^	0.009	0.019
Empty weight (g) **						
Crop	6.53 ^Aa^	5.46 ^Bc^	6.24 ^ab^	5.78 ^bc^	0.126	0.013
Proventriculus	5.89 ^AB^	5.50 ^B^	6.09 ^A^	5.84 ^AB^	0.067	0.009
Gizzard	23.65 ^ab^	22.17 ^b^	24.72 ^a^	22.64 ^ab^	0.316	0.023
*Length (cm)*						
Duodenum	25.44	25.29	25.53	24.76	0.149	0.636
Jejunum	72.30	69.19	69.97	70.02	0.502	0.180
Ileum	69.58	68.98	71.38	67.14	0.678	0.235
Ceca	33.32 ^a^	33.32 ^a^	35.19 ^A^	30.90 ^Bb^	0.397	0.001
Large intestine	6.28 ^b^	5.69 ^Bb^	7.68 ^Aa^	6.82 ^ab^	0.198	0.001
Total length of intestines	206.93 ^ab^	202.46 ^ab^	209.76 ^a^	199.64 ^b^	1.341	0.050

Means in the same row with different superscripts A, B significant at *p* < 0.01; a, b, c at *p* < 0.05. * One-factorial ANOVA. ** Analysis of covariance.

**Table 4:** Number of microorganisms in crop and ileum.

Treatment	Control	Wheat	Barley	Triticale	SEM	*p*-Value
Crop						
TAMC	6.34 ^A^	4.11 ^D^	4.63 ^C^	5.26 ^B^	0.162	0.000
*E. coli*	0.00 ^B^	1.78 ^Aa^	0.60 ^b^	0.00 ^B^	0.183	0.000
*Lactobacillus* sp.	3.35 ^A^	2.51 ^C^	2.22 ^C^	2.93 ^B^	0.088	0.000
TYMC	2.39 ^A^	2.21 ^AB^	2.05 ^B^	2.25 ^AB^	0.033	0.002
Ileum						
TAMC	4.11	4.15	4.09	4.16	0.015	0.384
*E. coli*	1.20 ^ab^	1.77 ^ab^	2.12 ^a^	0.78 ^b^	0.190	0.046
*Lactobacillus* sp.	2.23 ^B^	2.26 ^B^	3.23 ^A^	2.43 ^B^	0.078	0.000
TYMC	1.75 ^ab^	0.45 ^b^	1.55 ^ab^	1.96 ^a^	0.167	0.002

Means in the same row with different superscripts A, B, C, D significant at *p* < 0.01; a, b at *p* < 0.05. TAMC—total aerobic microbial count, TYMC—total yeast and mold count.

**Table 5:** Correlation between weight of organs in foregut or length of intestine and count of individual microorganisms in crop and ileum (n = 2.8).

Item	Crop				Ileum			
	TAMC	*E. coli*	*Lactobacillus* sp.	TYMC	TAMC	*E. coli*	*Lactobacillus* sp.	TYMC
*Empty weight*								
Crop	0.467	−0.490	ns	ns	ns	ns	ns	ns
Proventriculus	ns	−0.406	ns	ns	ns	ns	0.482	ns
Gizzard	ns	ns	ns	ns	ns	ns	0.490	ns
*Length*								
Duodenum	ns	ns	ns	ns	ns	ns	ns	−0.391
Jejunum	0.401	ns	ns	ns	ns	ns	ns	ns
Ileum	ns	ns	ns	−0.418	ns	ns	ns	ns
Ceca	ns	ns	−0.407	−0.456	ns	ns	0.449	ns
Large intestine	ns	−0.425	ns	−0.430	ns	ns	0.613	ns
Total length of intestines	ns	ns	ns	ns	ns	ns	ns	ns

ns—non-significant correlation between variables (*p* > 0.05).

## Data Availability

The raw data supporting the conclusions of this article will be made available by the authors on request.
